# Cardiovascular Organoids With Adjustable Endothelial Composition via SOX17‐Engineered hPSCs

**DOI:** 10.1002/bit.70275

**Published:** 2026-06-21

**Authors:** Po‐Yu Liang, Gyuhyung Jin, Nathan R. Petrucci, Xiaojun Lance Lian, Xiaoping Bao

**Affiliations:** ^1^ Davidson School of Chemical Engineering Purdue University West Lafayette Indiana USA; ^2^ Purdue Institute for Cancer Research West Lafayette Indiana USA; ^3^ Weldon School of Biomedical Engineering Purdue University West Lafayette Indiana USA; ^4^ Department of Biomedical Engineering The Pennsylvania State University University Park Pennsylvania USA; ^5^ The Huck Institutes of the Life Sciences The Pennsylvania State University University Park Pennsylvania USA; ^6^ Department of Biology The Pennsylvania State University University Park Pennsylvania USA

**Keywords:** cardiomyocyte, cardiovascular disease, genome editing, human pluripotent stem cell, organoid, SOX17

## Abstract

Organoids are considered a novel modeling platform for studying human biology and advancing health research. With the ability to demonstrate complex 3D structure and multicellular interactions, organoids have advanced studies in all major organs as a reliable model. In this study, we generated an advanced cardiovascular organoid by using a genome‐edited human pluripotent stem cell line with inducible *SOX17* expression, enabling controlled endothelial specification, adjustable cell‐type composition, and human heart‐like morphology. Our organoids recapitulated the cardiotoxic phenotypes of FDA‐approved chemotherapeutic doxorubicin, manifesting as decreased cell viability and diminished contractile activity. Cryoinjury‐induced myocardial infarction in our organoids led to reduced beating, viability, and α‐actinin expression, along with increased fibroblast formation, which were mitigated by Captopril. Lastly, isoproterenol treatment increased peak Ca^2+^ transient amplitude and shortened APD_50_ in our organoids, consistent with previously reported β‐adrenergic responses. In summary, we established a protocol for generating in vitro 3D cardiovascular organoids with controllable cellular composition and heart‐like structures, providing a robust and easy‐to‐produce platform for future studies of human heart disease.

## 1 Introduction

Cardiovascular diseases cause a great challenge to global health care system as the leading cause of death around the world (Murphy 2024; Rossi et al. 2021; Sallam et al. 2022). Establishing a reliable and reproducible in vitro cardiovascular model has been crucial for cardiovascular study (Dardano et al. 2024; Eisen et al. 2019; Halloin et al. 2019; Hofbauer et al. 2021; Knight et al. 2021; Volmert et al. 2023; Yi et al. 2021). In the past few years, many platforms have been established to create 3D cardiac models, such as 3D printing organoids (Kupfer et al. 2020; Radhakrishnan et al. 2025; Ye et al. 2024), optogenetics‐ or biomaterials‐mediated patterning (Hellwarth et al. 2021; Hoang et al. 2021; Repina et al. 2020; Repina et al. 2023), heart‐on‐a‐chip system (Butler and Reyes 2024; Kieda et al. 2024; Landau et al. 2024; Mozneb et al. 2024), and embryoid body differentiation of cardiovascular organoids (Ergir et al. 2022; Schmidt et al. 2023; Song et al. 2024). However, recapitulating two key features of cardiogenesis when differentiating in vitro 3D cardiac models remains a significant challenge: (1) Specific cardiovascular cell type composition; (2) Intricate intercellular interaction (Farah et al. 2024; Giacomelli et al. 2017; Giacomelli et al. 2020; Qu et al. 2022; Voges et al. 2023; Yang et al. 2022).

Previously, we reported a protocol for 3D vascularized cardiac organoids (vCO) with a chamber‐like structure (Liang et al. 2022). While the model shares similar features to the human heart, the ratio between cardiomyocytes (CMs) and endothelial cells (ECs) could not be precisely modulated. To solve the problem, we investigated gene‐mediated vascularization in cardiovascular organoids. SOX17 has been identified as a critical regulator of cardiogenesis, promoting endothelial differentiation in both human pluripotent stem cells (hPSCs) and mouse embryos (Liu et al. 2014; Ream et al. 2024; Saba et al. 2019). To make reproducible heart‐like cardiac organoids (hCOs), we utilized CRISPR/Cas9 technology to construct a doxycycline‐inducible SOX17 hPSC line and demonstrated that induced overexpression of SOX17 at cardiac progenitor stage leads to reproducible EC generation within our 3D cardiac organoids (COs). By combining the SOX17‐edited and unedited hPSCs, we developed a one‐step protocol for generating endothelialized COs in a reproducible manner. Our hCOs displayed organized morphology and diverse cell types resembling those of the human heart (Protze et al. 2019), providing a valuable platform for future cardiovascular research.

### 1.1 Material and Methods

Key Resources Table:

**Table:** 

Reagent	Source	Identifier
Antibodies		
SOX17‐APC (1:50)	R&D Systems	Cat#IC1924A
CD31‐FITC (1:100)	Miltenyi Biotec	Cat#130‐110‐668
NKX2.5	Invitrogen	Cat# PA5‐85768
VE‐cadherin (1:50)	Santa Cruz	Cat#sc9989
cTnT	Abcam	Cat#ab91605
WT1	ThermoFisher Scientific	Cat#PA5‐18548
MYL2	Proteintech	Cat#10906‐1‐AP
MYL7	Invitrogen	Cat# MA5‐26390
VIM	R&D Systems	Cat#Af2105
Propidium iodide (PI)	BD Pharmingen	Cat#556463
ACTN	Invitrogen	Cat#ab68167
Fluo‐4, AM	Invitrogen	Cat#F14201
Chemicals, peptides, and proteins		
Matrigel	Corning	Cat#3554277
iMatrix‐511	Iwai North America Inc	Cat#N‐892021
mTeSR™ Plus	STEMCELL Technologies	Cat#100‐0276
RPMI 1640	Gibco	Cat#11875093
B‐27™ Supplement (50X), serum free	Gibco	Cat#17504044
CellTiter‐Glo 2.0 Assay	Promega	Cat#G9241
MethoCult H4434	STEMCELL Technologies	Cat#04434
EDTA	Thermo Fisher Scientific	Cat#15575020
Y‐27632	Cayman Chemical	Cat#10005583
CHIR99021	Cayman Chemical	Cat#13122
Ascorbic acid (L‐ascorbic acid 2‐phosphate)	Sigma‐Aldrich	Cat#A8960
Bovine serum albumin	Sigma‐Aldrich	Cat#A1923
Human VEGF	PEPROTECH	Cat#100‐20
16% Formaldehyde	Thermo Fisher Scientific	Cat#28906
Triton X‐100	Thermo Fisher Scientific	Cat#A16046. AE
Blotting‐grad blocker (dry milk)	Bio‐Rad Laboratories, Inc	Cat#1706404
Hoechst33342	Invitrogen	Cat#AM‐105
Dulbecco's phosphate‐buffered saline	Sigma‐Aldrich	Cat#D8537
Doxorubicin hydrochloride	Sigma‐Aldrich	Cat#25316‐40‐9
Isoproterenol hydrochloride	Thermo Fisher Scientific	Cat#51‐30‐9
Captopril	Thermo Fisher Scientific	Cat#62571‐86‐2
Experiment models: Cell lines		
Human pluripotent stem cell: H9	WiCell	RRID:CVCL_9773
Software and algorithms		
ImageJ	Schindelin et al.	https://imagej.net/ij/
Other		
Sterile biosafety cabinets	Thermo Fisher Scientific	Cat#1323TS
Humidified tissue culture incubator	Thermo Fisher Scientific	Cat#51026282
Leica DMi8 microscope	Leica Microsystems	Cat# 12‐550‐15
Zeiss LSM 900 confocal microscope	ZEISS	

### 1.2 Methods

#### 1.2.1 Maintenance of Human Pluripotent Stem Cells

H9 cell lines were purchased from WiCell and cultured in mTeSR Plus medium on Matrigel‐coated plates at 37°C in humidified atmosphere with 5% CO_2_. Cell passaging was conducted using 0.1 mM EDTA when cells reached 80% ~ 90% confluency and replated in mTeSR plus medium with 5 μM Y27632 (Lian et al. 2014).

#### 1.2.2 Hanging Drop Preparation for Embryoid Body Formation

For embryoid body (EB) formation, 20 μL droplets were formed by pipetting on the inner side of a 96‐well U‐bottom culture plate lid. Droplets contained 100,000 hPSCs/mL and were composed of mTeSR Plus supplemented with 5 μM Y‐27632 (Cayman Chemical) and iMatrx‐511 (1:100). 100 μL Phosphate‐Buffered Saline (PBS) was added to each well before returning the lid to culture plate. After 24 h, EBs were transferred with a pipette to a new 96‐well U‐bottom culture plate (single organoid per well) and cultured in mTeSR Plus with 5 μM Y‐27632 for 1 day. The resulting EBs were then ready for further differentiation (Banerjee and Bhonde 2006; Wang and Yang 2008).

#### 1.2.3 Cardiovascular Organoid Differentiation

Rested EBs were treated with 6 μM CHIR99021 (Cayman Chemical) in RPMI 1640 medium for 48 h at day 0. On day 3, medium was replaced with RBL medium (0.1056 g L‐ascorbic acid 2‐phosphate and 0.25 g Bovine Serum Albumin (BSA) in 500 ml RPMI 1640 medium) containing 2 μM Wnt‐C59. On day 4, the medium was replaced with RBL medium. For cardiomyocyte organoid differentiation, RPMI 1640 medium supplemented with 1× B27 (RPMI + B27) was added on day 6. Starting on day 7, the medium was changed daily to RPMI + B27 supplemented with 3 μM doxycycline (DOX) until day 10. After day 10, organoids were maintained in RPMI + B27, with the medium replaced every 3 days. For cardiovascular organoid differentiation, RPMI + B27 with 3 μM CHIR99021 was added on Day 6. Starting on Day 7, the medium was changed daily to RPMI + B27 supplemented with 2 μM DOX until Day 10. After day 10, organoids were maintained in RPMI + B27, with the medium replaced every 3 days.

#### 1.2.4 Immunostaining and Imaging

Differentiated organoids were fixed in PBS −/− containing 4% formaldehyde for 90 min at 4°C. Following fixation, the solution was aspirated, and organoids were washed twice with PBS. Organoids were incubated overnight at 4°C with the appropriate primary antibodies (1:50) in PBS containing 0.1% Triton X and 5% BSA supplemented with 5% nonfat dry milk. After washing organoids with PBS twice, organoids were stained with secondary antibody (1:50) in PBS containing 0.1% Triton X and 5% BSA at 4°C for 2 h. Hoechst 33342 (2′‐(4‐ethoxyphenyl)−5‐(4‐methylpiperazin‐1‐yl)−2,5′‐bibenzimidazole) (16.2 μM) was added to the solution at 1:1000 ratio during the final 30 min of incubation. After 2 h, staining medium was aspirated, and organoids were washed with PBS twice. Organoids were imaged using a Leica DMi8 microscope or a Zeiss LSM 900 confocal microscope. The images were processed using ImageJ.

#### 1.2.5 Doxorubicin (DXR) Toxicity Test

DXR stock solution (5 mM) was prepared in DMSO. The toxicity solution was prepared by diluting DXR in RPMI + B27 at final dilution ratios of 1:1000 or 1:5000. Organoids were exposed to the toxicity solution for 3 days, replacing the solution daily. Following the 3‐day treatment, organoids were maintained in RPMI + B27.

#### 1.2.6 Cell Viability Assay

CellTiter‐Glo 2.0 Assay was conducted by following Promega Corporation CellTiter‐Glo 2.0 Assay protocol. 50 μL of RPMI + B27 and 50 μL CellTiter‐Glo reagent was added to organoids. Organoids were incubated on a shaking incubator for 2 min followed by 10 min under room temperature. Luminescence was recorded using plate reader (Molecular Devices; San Jose, CA, SpectraMax iD3).

#### 1.2.7 Myocardial Infarction (MI) Model

A MI model was established by inducing cryoinjury in cardiovascular organoids. Cryoinjury was induced using a sterile needle chilled in liquid nitrogen for 1 min. After removing the RPMI + B27 with insulin medium from the cardiovascular organoids, the frozen needle was applied directly to the organoids for 5 s. Fresh RPMI + B27 with insulin medium, with or without captopril (CAP), was then added for recovery culture.

#### 1.2.8 Cryosection and Immunofluorescence

Organoids were rinsed in PBS twice and fixed in 4% paraformaldehyde solution overnight at 4°C. After washing with PBS three times, organoids were incubated in 30% sucrose at 4°C overnight. Organoids were subsequently embedded in optimal cutting temperature (O.C.T) compound and frozen at −80°C until cryosectioning. Frozen organoids were sliced in 50 μM sections and collected on glass slides. The cryosection was washed three times with PBS and incubated overnight at 4°C with the appropriate primary antibodies (1:50) in PBS containing 0.1% Triton X and 5% BSA supplemented with 5% nonfat dry milk. After washing organoids with PBS twice, organoids were stained with secondary antibody (1:50) in PBS containing 0.1% Triton X and 5% BSA at 4°C for 2 h. Hoechst 33342 (2′‐(4‐ethoxyphenyl)−5‐(4‐methylpiperazin‐1‐yl)−2,5′‐bibenzimidazole) (16.2 μM) was added to the solution at 1:1000 ratio during the final 30 min of incubation. After 2 h, staining medium was aspirated, and organoids were washed with PBS twice. Organoids were imaged using a Leica DMi8 microscope or a Zeiss LSM 900 confocal microscope.

#### 1.2.9 Isoproterenol (ISO) Model Analysis

ISO stock solution (100 mM) was prepared in PBS. Working ISO medium was generated by diluting the stock 1:1000 in RPMI + B27. Organoids were treated with ISO‐containing medium for 3 days, with a daily medium change. Following treatment, organoids were maintained in RPMI + B27.

#### 1.2.10 Membrane Calcium Voltage Analysis

Calcium voltage staining was conducted by following Invitrogen standard Fluo‐A AM protocol. The labeling solution was prepared by dissolving 50 μg of Fluo‐4 AM in 50 μL of DMSO. The staining solution was prepared by diluting the labeling solution 1:200 in RPMI + B27 with insulin. Beating organoids were incubated with the staining solution for 40–60 min at 37°C in the dark. Following incubation, organoids were gently washed twice with PBS and transferred to fresh RPMI + B27 with insulin medium for imaging. Calcium transients were recorded within 30 min after staining using a microscope equipped with a FITC channel. After recording, the intensity of FITC was quantified by ImageJ. The amplitude is measured by dividing the maximum intensity to the lowest intensity in one beat. A representative recording of calcium activity is shown in Video S1.

#### 1.2.11 Viability Analysis

Organoids were incubated with propidium iodide (1:100 in PBS) for 30 min at room temperature. During the final 10 min of incubation, Hoechst 33342 was added at a 1:1000 dilution. After staining, the solution was aspirated, and organoids were washed twice with PBS. Organoids were then imaged by fluorescence microscopy, and cell numbers were quantified using ImageJ.

## 2 Results

### 2.1 Construction of AAVS1 XLone‐SOX17 hPSCs

**Figure 1:**
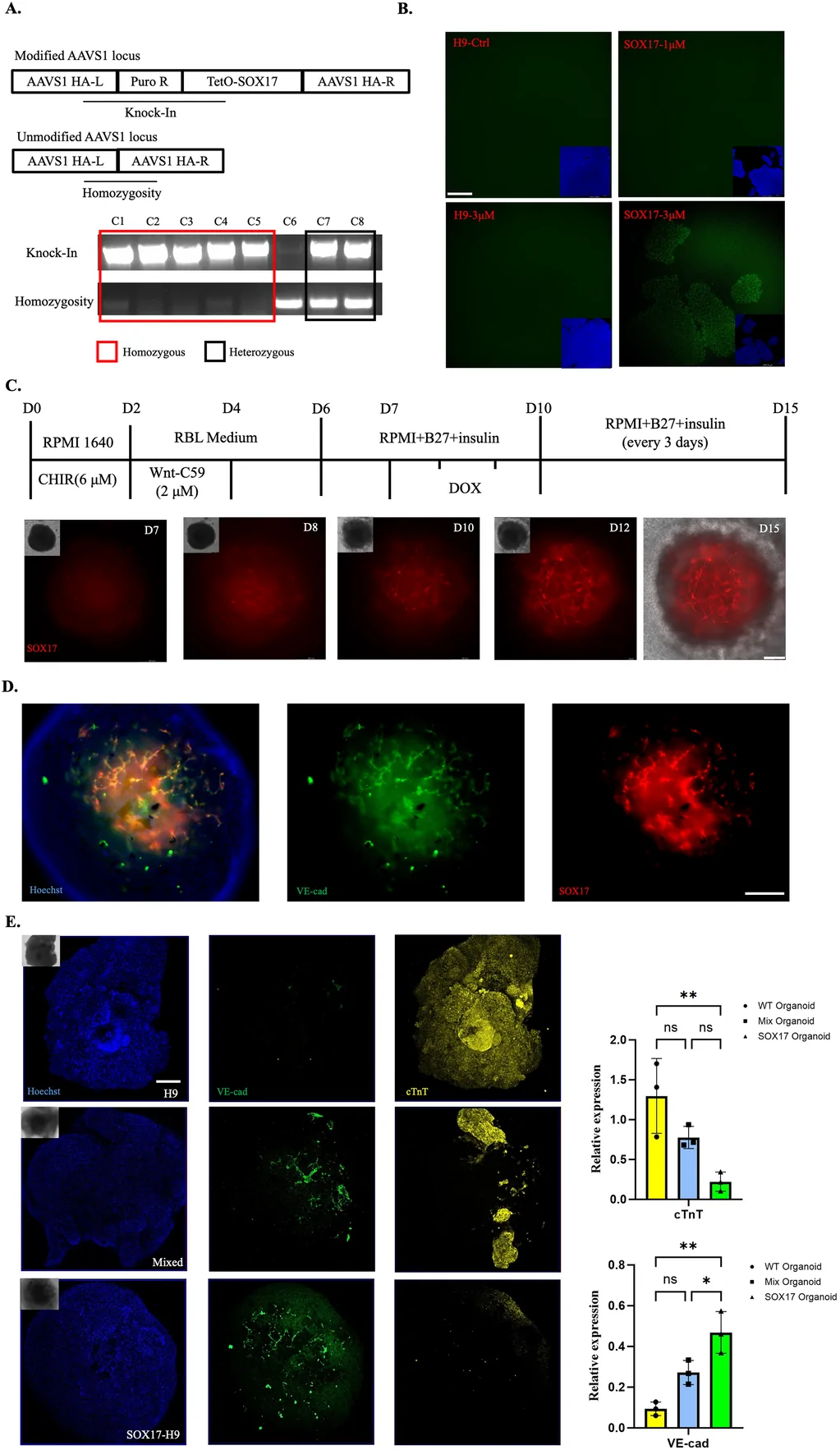
Endothelialized structure in hPSC‐derived cardiac organoids by *SOX17* overexpression. (A) Schematic of the *AAVS1* safe‑harbor knock‑in strategy and PCR genotyping of edited clones XLone‐SOX17 H9. Five clones were homozygous and two were heterozygous for the knock‑in. (B) Verification of SOX17 overexpression under doxycycline of *AAVS1* safe‑harbor knock‑in XLone‐SOX17 H9. Scale bar = 200 μM. (C) A protocol of differentiating embryoid bodies into cardiac organoids and representative brightfield images showing the emergence of SOX17^+^ endothelial networks between Day 7 and Day 15. Red = SOX17. Scale bar = 200 μM. (D) Representative immunofluorescence images demonstrating endothelialized SOX17^+^ structures and co‐expression of VE‐cadherin and SOX17 within cardiac organoids. Green = VE‐cadherin, red = SOX17, blue = Hoechst. Scale bar = 100 μM. (E) Representative immunofluorescence images and quantification of VE‐cadherin, cTnT, and Hoechst of organoids with different H9 to SOX17 cell line ratios (1:0, 2:1, 0:1). Green = VE‐cadherin, yellow = cTnT, blue = Hoechst. Scale bar = 200 μM. *n* = 3 independent organoids per condition. Data presented as normalized to Hoechst. Values = mean ± standard deviation (SD), one‐way ANOVA.

To control endothelial differentiation in our organoid system (Liu et al. 2024), we generated an H9 cell line with an *AAVS1*‐targeted, doxycycline (DOX)‐inducible SOX17 overexpression construct (SOX17‐H9) to enable inducible SOX17 expression during differentiation. Successful *AAVS1* insertion in H9 cells was confirmed by genotyping following drug selection (Figure 1A). One of the homozygous clones C3 was expanded and used for subsequent experiments. DOX was tested at concentrations ranging from 1 to 7 μM in our inducible SOX17 H9 cell line to minimize DOX‐associated toxicity while ensuring robust SOX17 expression (Ahler et al. 2013). While 1 μM DOX failed to induce SOX17 expression, treatment with 3 μM DOX activated SOX17 in the SOX17 H9 cell line, as confirmed by SOX17 immunostaining (Figure 1B, Supporting Figure 1). To minimize DOX‐associated toxicity during differentiation, we used 3 μM DOX in all subsequent experiments. We applied CM differentiation protocol for hPSCs (Lian et al. 2015; Mehta et al. 2014) to our SOX17‐H9 EBs with the addition of DOX during cardiac progenitor stage between Day 7 to Day 10 to initiate EC differentiation (Figure 1C). NKX2.5 expression on day 6 confirmed the differentiation of EBs into cardiac progenitor cells (Supporting Figure 2). SOX17 signal gradually emerged from day 8 and eventually formed a clear vascular structure around day 12 (Figure 1C) while no spontaneous beating occurred. The vascular structure located at the center of our organoids likely reflects their self‐organizing properties. Based on the observed vascular structures, we hypothesized that SOX17 was expressed in ECs, which was confirmed by co‐staining with VE‐cadherin and SOX17 in the resulting organoids (Figure 1D). Co‐expression of SOX17 and VE‐cadherin indicates that SOX17 can serve as a marker of endothelial cells, consistent with previous studies (Corada et al. 2013; Kim et al. 2023). To test the potential of orchestrating the ratio of ECs in COs, we cultured EBs with different H9 to SOX17‐H9 ratios (1:0; 2:1; 0:1) (Supporting Figure 3). After differentiation, H9 COs mainly showed cTnT signal with little VE‐cadherin expression while SOX17‐H9 COs demonstrated great vascular VE‐cadherin signal with no cTnT expression (Figure 1E). By combining both cell lines, we successfully created COs with both endothelial networks and beating CMs. The endothelial signal appeared to correlate with the initial SOX17‐H9 ratio (Figure 1E), suggesting that the number of endothelial cells in COs may be tunable. To generate organoids exhibiting both spontaneous beating and endothelial differentiation, we used a 2:1 mixture of H9 and SOX17‐H9 cells for all subsequent experiments.

### 2.2 Generation of Multicellular Cardiac Organoids

**Figure 2:**
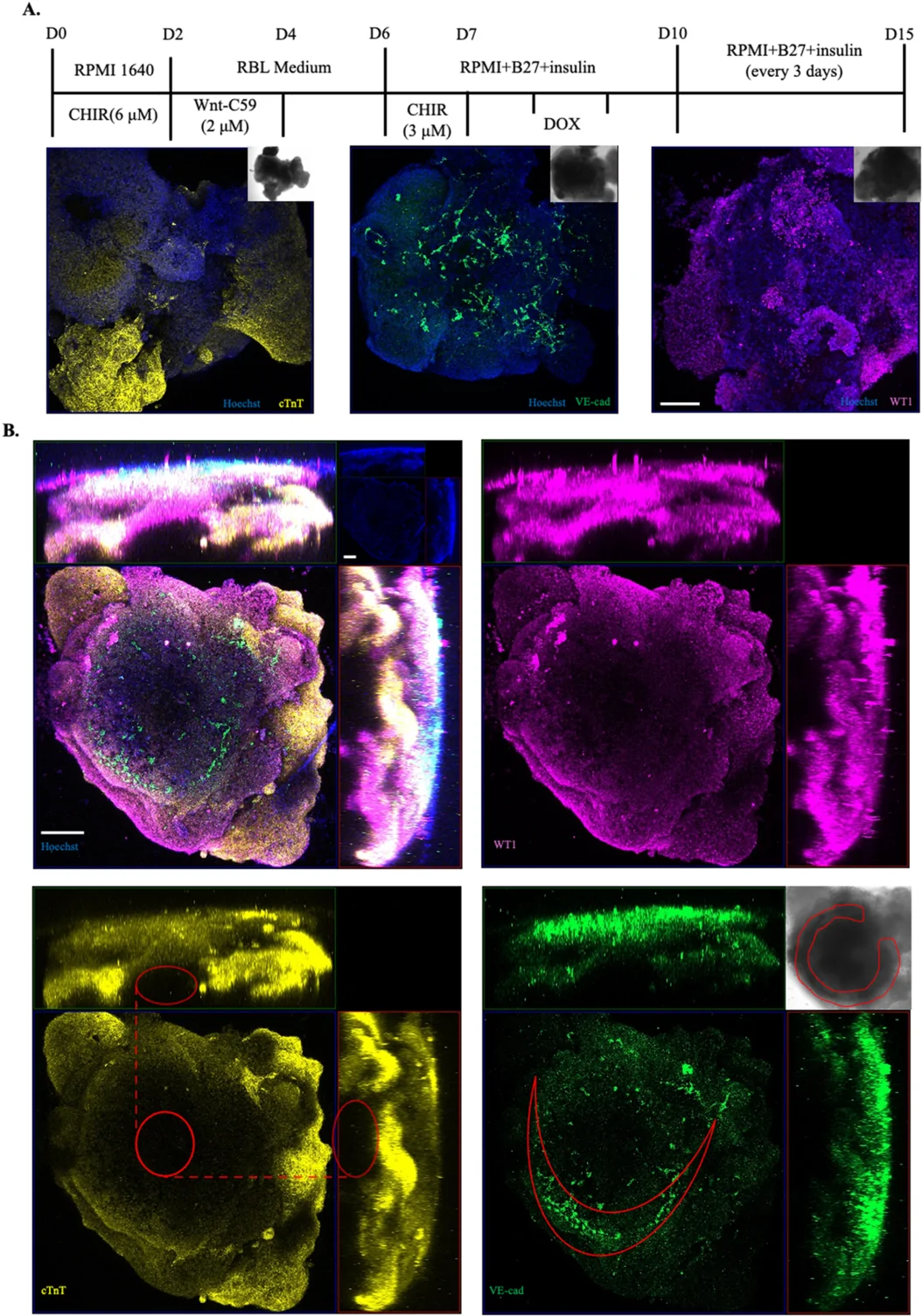
One‐step differentiation of cardiovascular organoids with major cardiac lineages and heart‐like morphology. (A) A protocol of differentiating cardiovascular organoids and representative immunofluorescence images of three major cardiovascular cell types within organoids. Green = VE‐cadherin, yellow = cTnT, purple = WT1, blue = Hoechst. Scale bar = 200 μM. (B) Representative confocal images of cardiovascular organoids demonstrating chamber formation, self‐organization, and the presence of major cardiovascular cell types. Green = VE‐cadherin, yellow = cTnT, purple = WT1, blue = Hoechst. Scale bar = 200 μM.

To generate cardiac organoids with three major cardiovascular cell types (ECs, CMs, and epicardial cells), we activated Wnt signaling pathway at cardiac progenitor stage to promote epicardial differentiation as previously reported (Bao et al. 2016; Bao et al. 2017; Branco et al. 2022b; Fernandes et al. 2023). On day 6, we added 3 μM CHIR to our organoids with RPMI + B27. After 1 day of Wnt activation, the medium was replaced with RPMI + B27 containing DOX and maintained for 3 days (Figure 2A). COs under new protocol preserved spontaneous beating and vascular SOX17 expression. The presence of each major cardiovascular cell type was verified by immunostaining (Figure 2A). To assess the morphology of our cardiovascular organoids, we co‐stained COs with major cardiovascular markers: VE‐cadherin for ECs, WT1 for epicardial cells, and cTnT for CMs (Figure 2B). Our COs showed consistent, lineage‐driven self‐organization, with internal lumen‐like cavities forming in 63.6% of samples (7/11) (Supporting Figure 4A). Immunofluorescence and cryosection analyses revealed clear spatial compartmentalization: ECs (SOX17^+^/VE‐cadherin^+^) were predominantly localized to the interior, lining the lumen‐like voids, whereas CMs and epicardial cells occupied the peripheral layers (Figure 2B, Supporting Figure 4B‐C). Co‐differentiation of cardiovascular cell types has been shown to promote CM maturation (Mikryukov et al. 2021; Saba et al. 2019). Although H9 COs expressed higher levels of the immature atrial‐associated marker MYL7, COs derived from mixed H9 and SOX17‐H9 cells showed reduced MYL7 expression accompanied by increased MYL2 expression (Supporting Figure 5). This shift in myosin light chain isoforms suggests a trend toward CM specification and maturation, potentially driven by cardiovascular intercellular interactions. Altogether, our cardiac organoids exhibit robust self‐organization, forming internal lumen‐like structures and a layered architecture, while displaying molecular features consistent with early CM subtype specification.

### 2.3 Chemotherapy Induced Cardiotoxicity Modeling

**Figure 3:**
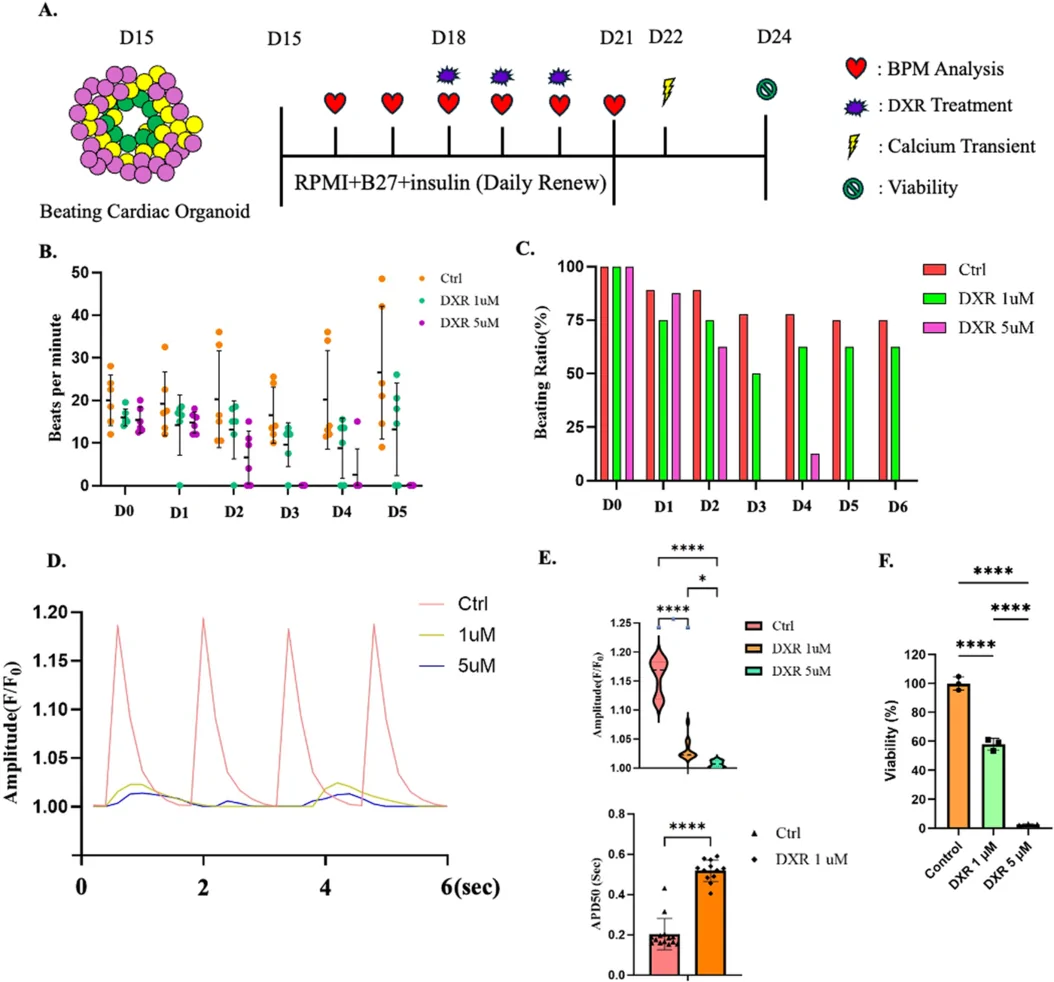
Advanced cardiovascular organoids simulate doxorubicin‐induced cardiotoxicity. (A) Schematic diagram of simulating and analyzing doxorubicin‐induced cardiotoxicity with cardiovascular organoids. (B) Time‐ and dose‐ dependent effects of doxorubicin on beating frequency of cardiovascular organoids. *n* = 6–8 independent organoids per condition. Values = mean ± SD. (C) Quantification of the percentage of visibly beating organoids under brightfield microscopy after treatment with different concentrations of doxorubicin. *n* = 8–13 independent organoids in each concentration. (D) Representative image of time‐lapse recordings of calcium transient activity in cardiovascular organoids following treatment with control (Ctrl), 1 μM, or 5 μM doxorubicin. (E) Quantification of amplitude (F/F_0_) and APD_50_ of calcium transient in cardiovascular organoids under different concentration (Ctrl, 1 µM, 5 μM) of doxorubicin treatment. *n* = 11–14 independent organoids per condition. Values = mean ± SD, Welch's *t*‐test. (F) Quantification of cardiovascular organoid viability following treatment with different concentrations (Ctrl, 1 μM, 5 μM) of doxorubicin. *n* = 11–14 independent organoids per condition. Values = mean ± SD, ordinary one‐way ANOVA.

Doxorubicin (DXR) is a widely used chemotherapy drug for cancer, which may cause irreversible heart damage (Burridge et al. 2016; Sheibani et al. 2022). To assess the extent to which our model recapitulates aspects of human heart biology, hCOs were treated with DXR under different concentration for 3 days. We measured the contractility and viability of hCOs after treatment to quantify the effect of DXR (Figure 3A). While the beating rate of each group remained in a certain range, DXR treatment significantly decreased the number of organoids which demonstrated spontaneous visible beating (Figure 3B). hCOs exposed to a higher DXR concentration (5 μM) eventually stop beating, whereas those treated with a lower dose (1 μM) retain measurable contractility (Figure 3C). We then analyzed the beating behavior of hCOs by measuring calcium transients. Although calcium transients were still detectable in hCOs treated with 5 μM DXR, their beating was markedly disrupted and failed to produce stable, consistent contractions (Figure 3D). Both calcium transient intensity and APD_50_ were significantly reduced under 5 μM DXR treatment (Figure 3E, Supporting Video 1,2), mirroring the cardiotoxic effects of DXR reported in human hearts (J. Yang et al. 2024). We then measured the viability of hCOs after 6 days of DXR treatment using Promega CellTiter‐Glo 2.0 kit. Treatment with 5 μM DXR resulted in more than 80% cell death in hCOs, although the organoids retained their spherical morphology (Figure 3F). These results suggest that hCOs may serve as a useful platform for drug screening.

### 2.4 In Vitro Modeling of Cardiac Injury

**Figure 4:**
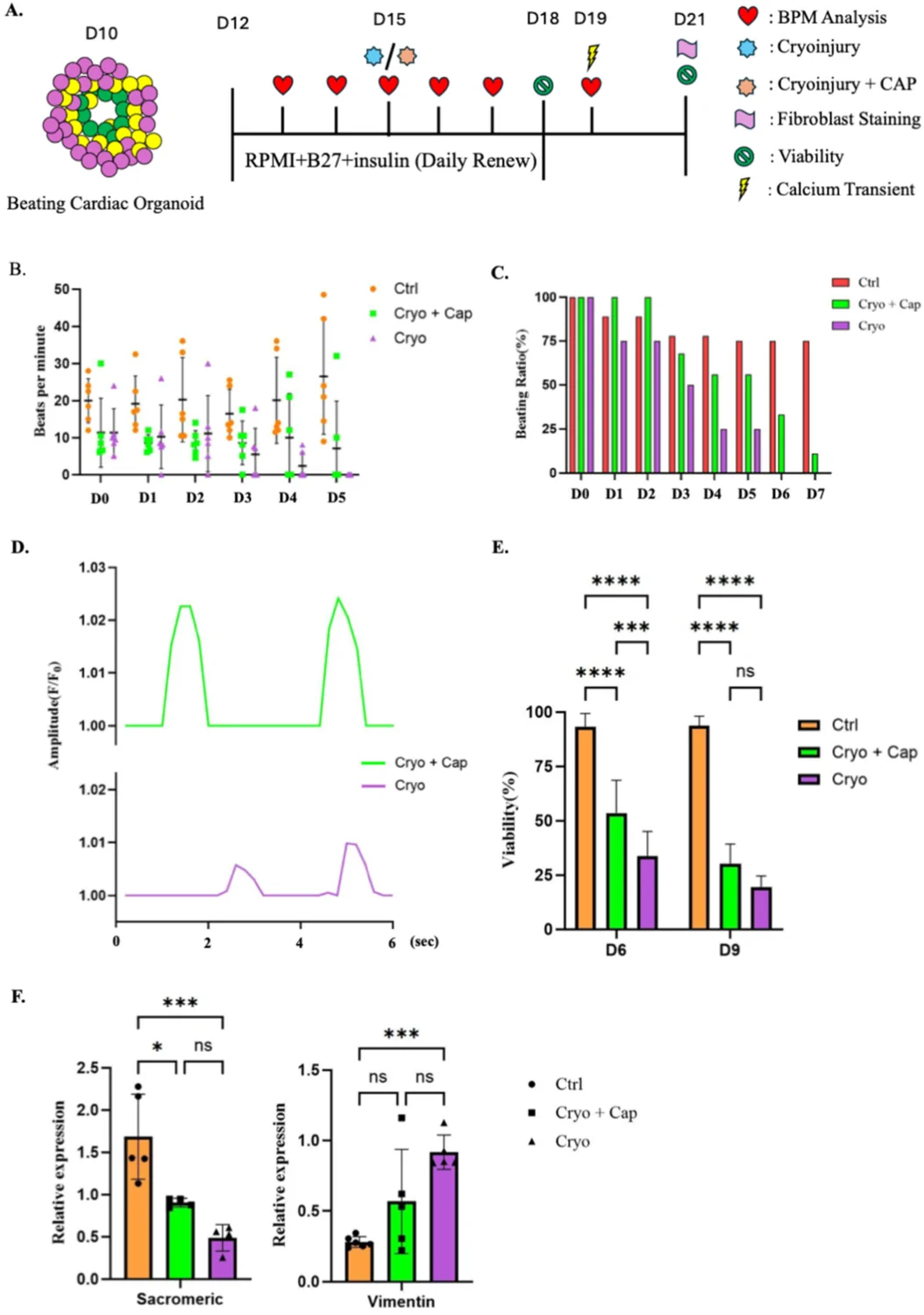
Advanced cardiovascular organoids model myocardial infarction by cryoinjury and captopril. (A) Schematic diagram of modeling and analyzing myocardial infarction with advanced cardiovascular organoids by cryoinjury. (B) Time‐dependent effects of cryoinjury and captopril on beating frequency of cardiovascular organoids. *n* = 6–8 independent organoids per condition. Values = mean ± SD. (C) Quantification of percentage of organoids visibly beating under brightfield microscopy under each condition (Ctrl, Cryo, Cryo + Cap). *n* = 8–13 independent organoids in each condition. (D) Representative images of time‐lapse recording of calcium transient activity in cardiovascular organoids after cryoinjury or cryoinjury plus captopril treatment. (E) Quantification of cardiovascular organoid viability under different conditions (Ctrl, Cryo, Cryo + Cap). *n* = 8–10 independent organoids per condition. Values = mean ± SD, two‐way ANOVA. (F) Quantification of sacromeric alpha actinin and VIMENTIN in organoids under each condition (Ctrl, Cryo, Cryo + Cap). *n* = 4–6 independent organoids per condition. Data presented as normalized to Hoechst. Values = mean ± SD, one‐way ANOVA.

To evaluate the utility of hCOs for disease modeling, we induced cryoinjury to simulate myocardial infarction (MI) (Kar et al. 2024; Richards et al. 2020). We induced cryoinjury on day 15 post‐differentiation (Figure 4A). We monitored the beating rate and ratio following cryoinjury and subsequently assessed calcium transients and viability to evaluate the extent of damage to hCOs (Figure 4A). Captopril (CAP), an FDA‐approved medication for myocardial infarction, was administered after cryoinjury to assess its ability to mitigate functional impairment. Beating activity was rapidly impaired following cryoinjury. In the absence of CAP, hCOs showed a marked decline in both beating rate and beating area, with contractions ceasing entirely by day 6 post‐injury (Figure 4B‐C). In contrast, CAP‐treated hCOs retained limited but irregular beating activity and demonstrated higher viability (Figure 4D,E). We then stained hCOs with a common muscle cell marker, sarcomeric alpha actinin, to assess CM integrity. hCOs treated with cryoinjury and CAP demonstrated higher sarcomeric alpha actinin intensity compared to hCOs treated with cryoinjury without CAP (Figure 4F). To validate if fibroblasts were differentiated from epicardial cells due to cryoinjury (Ciucci et al. 2023), hCOs were stained with VIMENTIN (VIM), a fibroblast marker, after treatment. hCOs subjected to cryoinjury alone exhibited elevated fibroblast signals, as evidenced by enhanced VIM expression, potentially indicating increased fibrosis. CAP treatment mitigated fibroblast formation under the same conditions (Xia et al. 2025) (Figure 4F). Taken together, these findings indicate that our hCOs may serve as a promising platform for modeling cardiovascular diseases.

**Figure 5:**
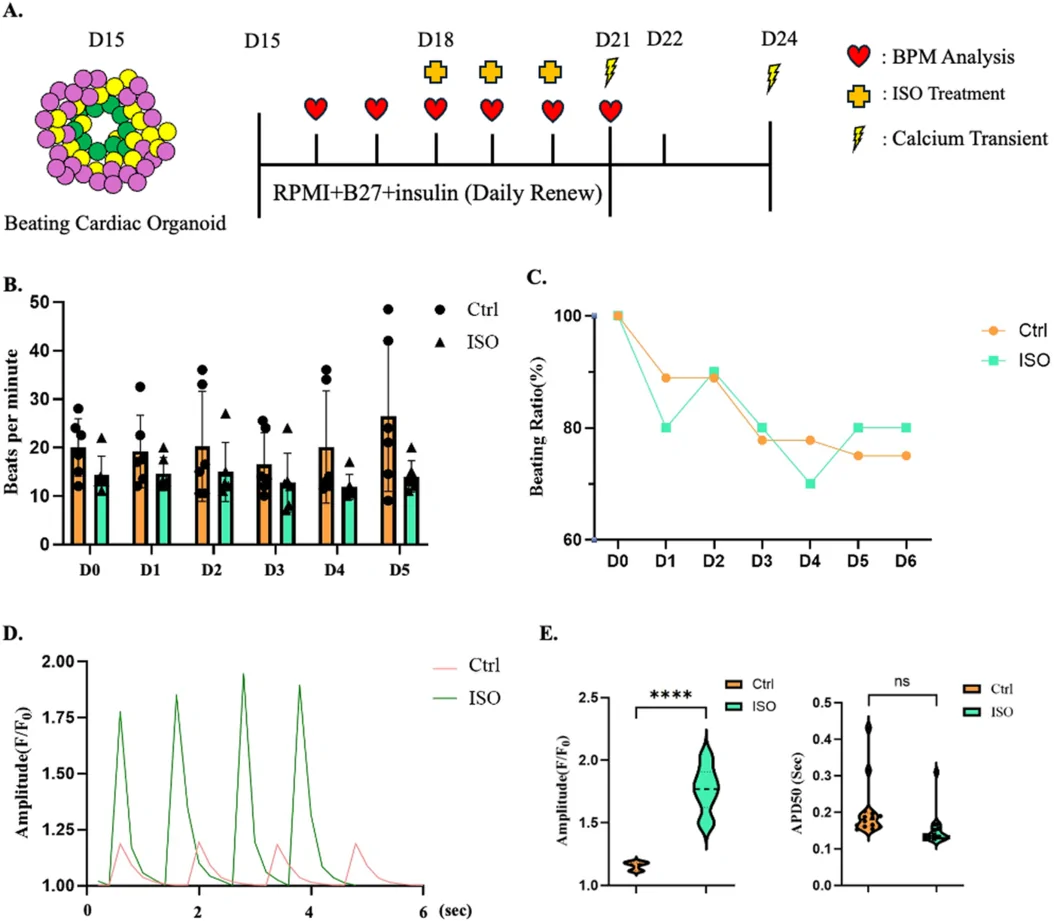
Advanced cardiovascular organoids as a platform for isoproterenol function assay. (A) Schematic diagram of mimicking isoproterenol's effect on human heart with advanced cardiovascular organoids by cryoinjury. (B) Time‐ and dose‐ dependent effects of isoproterenol on beating frequency of cardiovascular organoids. *n* = 6–8 independent organoids per condition. Values = mean ± SD. (C) Quantification of percentage of visibly beating organoids under brightfield microscopy with or without isoproterenol. *n* = 6–8 independent organoids in each concentration. (D and E) Representative images of time‐lapse recording of calcium transient activity in cardiovascular organoids after isoproterenol treatment (D) and the quantification of amplitude (F/F_0_) and APD_50_ (E) are shown. *n* = 10–14 independent organoids per condition. Values = mean ± SD, Welch's *t*‐test.

Isoproterenol (ISO) is a drug commonly used to treat bradycardia condition (Ebihara et al. 2017). We aimed to investigate if our hCOs can recapitulate human hearts’ response toward ISO. hCOs were treated with ISO for 3 days (Figure 5A). Following ISO treatment, hCOs exhibited an increased beating amplitude (Video S3) while maintaining a similar beating frequency compared to pre‐treatment levels (Figure 5B,C). To evaluate the effect of ISO on contractile behavior, calcium transient assays were performed on hCOs. ISO treatment significantly increased the peak amplitude (from 1.14 to 1.67) and shortened APD_50_ (Figure 5D,E). These findings were consistent with previous reports (Gardner et al. 1990) and further supported the utility of our hCOs as a platform for evaluating the effects of drugs on human hearts.

## 3 Discussion

While *ETV2* is an established master regulator that initiates stem cell differentiation toward endothelial lineages and is transiently expressed in early mesoderm (Wang et al. 2020), *SOX17* acts later in development to specify arterial identity and stabilize the endothelial phenotype (Grath and Dai 2024; Robinson et al. 2014). To better mimic the temporal hierarchy of embryonic development, we therefore employed *SOX17* to drive endothelial cell specification from cardiac progenitors within the organoids. Expression of *SOX17* during cardiac progenitor stage stimulated endothelial differentiation in our cardiac organoids, thereby facilitating controlled adjustment of cardiovascular cell type ratios to better mimic the human heart. By combining varying proportions of unedited and genome‐edited cell lines, we successfully generated organoids with tunable levels of vascularized ECs. By co‐differentiating multiple cardiovascular cell types, the intricate paracrine interplay between different cells may be retained, as partially demonstrated by improved CM maturation. Our COs exhibited robust self‐organization and developed 3D morphological features, including internal lumen‐like cavities and a layered architecture reminiscent of the human heart. This structural organization may reflect, in part, the stabilizing role of *SOX17*, which functions during development to refine endothelial identity and promote arterial fate. The platform showed strong potential for cardiovascular disease modeling, as demonstrated by human heart‐like physiological responses to isoproterenol and doxorubicin (Tatekoshi et al. 2024). Overall, our study indicates that *SOX17* overexpression at the cardiac progenitor stage efficiently promotes endothelial specification, providing an adaptable strategy for generating organoids with a tunable endothelial cell composition. This approach may be broadly useful for complex disease modeling and therapeutic development in cardiovascular research (Ng et al. 2021; Wauchop et al. 2023).

Although our organoids show considerable potential as a platform for cardiovascular research, several limitations remain. Our cardiovascular organoids comprised the three major cardiac cell types—ECs, CMs, and epicardial cells—but also contained minor, undesired cell populations, likely due to the inherent heterogeneity of stem cell differentiation (Cheng et al. 2021; Misfeldt et al. 2009; Moriwaki et al. 2023; Pinto et al. 2016; Silva et al. 2021). To develop a model that more closely resembles the human heart, these minor cell types cannot be overlooked. Technically, the dense 3D extracellular matrix and low total cell numbers per organoid also make it difficult to generate a high‐quality single‐cell suspension for accurate quantification of cellular composition by flow cytometry or other assays. Another limitation of this study was the lack of detailed elucidation of intercellular interactions (Lee et al. 2020; Nakajima et al. 2019; Odiete et al. 2012; Palencia‐Desai et al. 2015; Quijada et al. 2021). Early communication between cardiovascular cell types plays a crucial role in cardiogenesis. Although our protocol preserved some degree of crosstalk among these cells, the specific mechanisms by which they influence one another warrant further investigation. Addressing these limitations provides opportunities and direction for future research.

## Author Contributions

Po‐Yu Liang, Xiaojun Lance Lian, and Xiaoping Bao designed research. Po‐Yu Liang, Gyuhyung Jin, and Nathan R. Petrucci, performed research. Po‐Yu Liang, Xiaojun Lance Lian, and Xiaoping Bao wrote the paper.

## Conflicts of Interest

The authors declare no conflicts of interest.

## Supporting information

Supporting Video 1

Supporting Video 2

Supporting Video 3

Supporting Video 4

Supporting File 1

## Data Availability

The data that support the findings of this study are available from the corresponding author upon reasonable request.

## References

[bit70275-bib-0001] Ahler, E. , W. J. Sullivan , A. Cass , et al. 2013. “Doxycycline Alters Metabolism and Proliferation of Human Cell Lines.” PLoS One 8, no. 5: e64561. 10.1371/journal.pone.0064561. 23741339 PMC3669316

[bit70275-bib-0004] Banerjee, M. , and R. R. Bhonde . 2006. “Application of Hanging Drop Technique for Stem Cell Differentiation and Cytotoxicity Studies.” Cytotechnology 51, no. 1: 1–5. 10.1007/s10616-006-9001-z. 19002889 PMC3449481

[bit70275-bib-0005] Bao, X. , V. J. Bhute , T. Han , T. Qian , X. Lian , and S. P. Palecek . 2017. “Human Pluripotent Stem Cell‐Derived Epicardial Progenitors Can Differentiate to Endocardial‐Like Endothelial Cells.” Bioengineering & Translational Medicine 2, no. 2: 191–201. 10.1002/btm2.10062. 29170757 PMC5675097

[bit70275-bib-0006] Bao, X. , X. Lian , T. A. Hacker , et al. 2016. “Long‐Term Self‐Renewing Human Epicardial Cells Generated From Pluripotent Stem Cells Under Defined Xeno‐Free Conditions.” Nature Biomedical Engineering 1, no. 1: 0003. 10.1038/s41551-016-0003. PMC5408455 28462012

[bit70275-bib-0007] Branco, M. A. , T. P. Dias , J. M. S. Cabral , P. Pinto‐do‐Ó , and M. M. Diogo . 2022. “Human Multilineage Pro‐Epicardium/Foregut Organoids Support the Development of an Epicardium/Myocardium Organoid.” Nature Communications 13, no. 1: 6981. 10.1038/s41467-022-34730-7. PMC9666429 36379937

[bit70275-bib-0008] Burridge, P. W. , Y. F. Li , E. Matsa , et al. 2016. “Human Induced Pluripotent Stem Cell–Derived Cardiomyocytes Recapitulate the Predilection of Breast Cancer Patients to Doxorubicin‐Induced Cardiotoxicity.” Nature Medicine 22, no. 5: 547–556. 10.1038/nm.4087. PMC5086256 27089514

[bit70275-bib-0009] Butler, D. , and D. R. Reyes . 2024. “Heart‐on‐a‐Chip Systems: Disease Modeling and Drug Screening Applications.” Lab on a Chip 24, no. 5: 1494–1528. 10.1039/D3LC00829K. 38318723

[bit70275-bib-0011] Cheng, L. , M. Xie , W. Qiao , et al. 2021. “Generation and Characterization of Cardiac Valve Endothelial‐Like Cells From Human Pluripotent Stem Cells.” Communications Biology 4, no. 1: 1039. 10.1038/s42003-021-02571-7. 34489520 PMC8421482

[bit70275-bib-0012] Ciucci, G. , K. Rahhali , G. Cimmino , et al. 2023. “Engineered Heart Tissue Maturation Inhibits Cardiomyocyte Proliferative Response to Cryoinjury.” Journal of Tissue Engineering 14: 20417314231190147. 10.1177/20417314231190147. 37842206 PMC10571691

[bit70275-bib-0013] Corada, M. , F. Orsenigo , M. F. Morini , et al. 2013. “Sox17 Is Indispensable for Acquisition and Maintenance of Arterial Identity.” Nature Communications 4, no. 1: 2609. 10.1038/ncomms3609. PMC3826640 24153254

[bit70275-bib-0014] Dardano, M. , F. Kleemiß , M. Kosanke , et al. 2024. “Blood‐Generating Heart‐Forming Organoids Recapitulate Co‐Development of the Human Haematopoietic System and the Embryonic Heart.” Nature Cell Biology 26, no. 11: 1984–1996. 10.1038/s41556-024-01526-4. 39379702 PMC11567889

[bit70275-bib-0015] Ebihara, T. , M. Morita , M. Kawada , K. Amano , F. Kato , and Y. Nakata . 2017. “Efficacy of Isoproterenol for Treating Amlodipine Overdose Resulting in Bradycardia.” Acute Medicine & Surgery 4, no. 3: 353–357. 10.1002/ams2.284. 29123890 PMC5674457

[bit70275-bib-0016] Eisen, B. , R. Ben Jehuda , A. J. Cuttitta , et al. 2019. “Electrophysiological Abnormalities in Induced Pluripotent Stem Cell‐Derived Cardiomyocytes Generated From Duchenne Muscular Dystrophy Patients.” Journal of Cellular and Molecular Medicine 23, no. 3: 2125–2135. 10.1111/jcmm.14124. 30618214 PMC6378185

[bit70275-bib-0017] Ergir, E. , J. O.‐D. La Cruz , S. Fernandes , et al. (2022). Generation and Maturation of Human iPSC‐Derived Cardiac Organoids in Long Term Culture. 10.1101/2022.03.07.483273. PMC9579206 36257968

[bit70275-bib-0018] Farah, E. N. , R. K. Hu , C. Kern , et al. 2024. “Spatially Organized Cellular Communities Form the Developing Human Heart.” Nature 627, no. 8005: 854–864. 10.1038/s41586-024-07171-z. 38480880 PMC10972757

[bit70275-bib-0019] Fernandes, I. , S. Funakoshi , H. Hamidzada , S. Epelman , and G. Keller . 2023. “Modeling Cardiac Fibroblast Heterogeneity From Human Pluripotent Stem Cell‐Derived Epicardial Cells.” Nature Communications 14, no. 1: 8183. 10.1038/s41467-023-43312-0. PMC10713677 38081833

[bit70275-bib-0020] Gardner, M. J. , D. E. Johnstone , R. D. Janes , G. A. Klassen , and J. A. Armour . 1990. “Effects of Increasing Heart Rate Induced by Efferent Sympathetic Neuronal Stimulation, Isoproterenol or Cardiac Pacing on Myocardial Function and Oxygen Utilization.” Pacing and Clinical Electrophysiology 13, no. 11: 1393–1400. 10.1111/j.1540-8159.1990.tb04014.x. 1701893

[bit70275-bib-0021] Giacomelli, E. , M. Bellin , L. Sala , et al. 2017. “Three‐Dimensional Cardiac Microtissues Composed of Cardiomyocytes and Endothelial Cells Co‐Differentiated From Human Pluripotent Stem Cells.” Development 144, no. 6: 1008–1017. 10.1242/dev.143438. 28279973 PMC5358113

[bit70275-bib-0022] Giacomelli, E. , V. Meraviglia , G. Campostrini , et al. 2020. “Human‐IPSC‐Derived Cardiac Stromal Cells Enhance Maturation in 3D Cardiac Microtissues and Reveal Non‐Cardiomyocyte Contributions to Heart Disease.” Cell Stem Cell 26, no. 6: 862–879.e11. 10.1016/j.stem.2020.05.004. 32459996 PMC7284308

[bit70275-bib-0023] Grath, A. , and G. Dai . 2024. “SOX17/ETV2 Improves the Direct Reprogramming of Adult Fibroblasts to Endothelial Cells.” Cell Reports Methods 4, no. 3: 100732. 10.1016//j.crmeth.2024.100732. 38503291 PMC10985233

[bit70275-bib-0024] Halloin, C. , K. Schwanke , W. Löbel , et al. 2019. “Continuous WNT Control Enables Advanced HPSC Cardiac Processing and Prognostic Surface Marker Identification in Chemically Defined Suspension Culture.” Stem Cell Reports 13, no. 2: 366–379. 10.1016/j.stemcr.2019.06.004. 31353227 PMC6700605

[bit70275-bib-0025] Hellwarth, P. B. , Y. Chang , A. Das , et al. 2021. “Optogenetic‐Mediated Cardiovascular Differentiation and Patterning of Human Pluripotent Stem Cells.” Advanced Genetics 2, no. 3: e202100011. 10.1002/ggn2.202100011. 36620431 PMC9744544

[bit70275-bib-0026] Hoang, P. , A. Kowalczewski , S. Sun , et al. 2021. “Engineering Spatial‐Organized Cardiac Organoids for Developmental Toxicity Testing.” Stem Cell Reports 16, no. 5: 1228–1244. 10.1016/j.stemcr.2021.03.013. 33891865 PMC8185451

[bit70275-bib-0027] Hofbauer, P. , S. M. Jahnel , N. Papai , et al. 2021. “Cardioids Reveal Self‐Organizing Principles of Human Cardiogenesis.” Cell 184, no. 12: 3299–3317.e22. 10.1016/j.cell.2021.04.034. 34019794

[bit70275-bib-0029] Kar, R. , D. Mukhopadhyay , and R. S. Angom . 2024. “Progress in Disease Modeling for Myocardial Infarction and Coronary Artery Disease: Bridging In Vivo and In Vitro Approaches.” Hearts 5, no. 4: 429–447. 10.3390/hearts5040031.

[bit70275-bib-0030] Kieda, J. , A. Shakeri , S. Landau , et al. 2024. “Advances in Cardiac Tissue Engineering and Heart‐On‐A‐Chip.” Journal of Biomedical Materials Research. Part A 112, no. 4: 492–511. 10.1002/jbm.a.37633. 37909362 PMC11213712

[bit70275-bib-0031] Kim, D. , A. Grath , Y. W. Lu , et al. 2023. “Sox17 Mediates Adult Arterial Endothelial Cell Adaptation to Hemodynamics.” Biomaterials 293: 121946. 10.1016/j.biomaterials.2022.121946. 36512862 PMC9868097

[bit70275-bib-0032] Knight, W. E. , Y. Cao , Y.‐H. Lin , et al. 2021. “Maturation of Pluripotent Stem Cell‐Derived Cardiomyocytes Enables Modeling of Human Hypertrophic Cardiomyopathy.” Stem Cell Reports 16, no. 3: 519–533. 10.1016/j.stemcr.2021.01.018. 33636116 PMC7940251

[bit70275-bib-0033] Kupfer, M. E. , W.‐H. Lin , V. Ravikumar , et al. 2020. “In Situ Expansion, Differentiation, and Electromechanical Coupling of Human Cardiac Muscle in a 3D Bioprinted, Chambered Organoid.” Circulation Research 127, no. 2: 207–224. 10.1161/CIRCRESAHA.119.316155. 32228120 PMC8210857

[bit70275-bib-0034] Landau, S. , Y. Zhao , H. Hamidzada , et al. 2024. “Primitive Macrophages Enable Long‐Term Vascularization of Human Heart‐On‐A‐Chip Platforms.” Cell Stem Cell 31, no. 8: 1222–1238.e10. 10.1016/j.stem.2024.05.011. 38908380 PMC11297673

[bit70275-bib-0035] Lee, J. , A. Sutani , R. Kaneko , et al. 2020. “In Vitro Generation of Functional Murine Heart Organoids via FGF4 and Extracellular Matrix.” Nature Communications 11, no. 1: 4283. 10.1038/s41467-020-18031-5. PMC7471119 32883967

[bit70275-bib-0036] Lian, X. , X. Bao , A. Al‐Ahmad , et al. 2014. “Efficient Differentiation of Human Pluripotent Stem Cells to Endothelial Progenitors via Small‐Molecule Activation of WNT Signaling.” Stem Cell Reports 3, no. 5: 804–816. 10.1016/j.stemcr.2014.09.005. 25418725 PMC4235141

[bit70275-bib-0037] Lian, X. , X. Bao , M. Zilberter , et al. 2015. “Chemically Defined, Albumin‐Free Human Cardiomyocyte Generation.” Nature Methods 12, no. 7: 595–596. 10.1038/nmeth.3448. 26125590 PMC4663075

[bit70275-bib-0038] Liang, P.‐Y. , Y. Chang , G. Jin , X. Lian , and X. Bao . 2022. “Wnt Signaling Directs Human Pluripotent Stem Cells Into Vascularized Cardiac Organoids With Chamber‐Like Structures.” Frontiers in Bioengineering and Biotechnology 10: 1059243. 10.3389/fbioe.2022.1059243. 36466327 PMC9715615

[bit70275-bib-0040] Liu, C. Z. , A. Prasad , B. Jadhav , et al. 2024. “Feeder‐Free Generation and Characterization of Endocardial and Cardiac Valve Cells From Human Pluripotent Stem Cells.” iScience 27, no. 1: 108599. 10.1016/j.isci.2023.108599. 38170020 PMC10758960

[bit70275-bib-0041] Liu, Y. , R. Kaneda , T. W. Leja , et al. 2014. “ *Hhex* and *Cer1* Mediate the Sox17 Pathway for Cardiac Mesoderm Formation in Embryonic Stem Cells.” Stem Cells 32, no. 6: 1515–1526. 10.1002/stem.1695. 24585688 PMC4260090

[bit70275-bib-0042] Mehta, A. , C. J. A. Ramachandra , G. L. Sequiera , et al. 2014. “Phasic Modulation of Wnt Signaling Enhances Cardiac Differentiation in Human Pluripotent Stem Cells by Recapitulating Developmental Ontogeny.” Biochimica et Biophysica Acta (BBA) ‐ Molecular Cell Research 1843, no. 11: 2394–2402. 10.1016/j.bbamcr.2014.06.011. 24978297

[bit70275-bib-0043] Mikryukov, A. A. , A. Mazine , B. Wei , et al. 2021. “BMP10 Signaling Promotes the Development of Endocardial Cells From Human Pluripotent Stem Cell‐Derived Cardiovascular Progenitors.” Cell Stem Cell 28, no. 1: 96–111.e7. 10.1016/j.stem.2020.10.003. 33142114

[bit70275-bib-0044] Misfeldt, A. M. , S. C. Boyle , K. L. Tompkins , V. L. Bautch , P. A. Labosky , and H. S. Baldwin . 2009. “Endocardial Cells Are a Distinct Endothelial Lineage Derived From Flk1+ Multipotent Cardiovascular Progenitors.” Developmental Biology 333, no. 1: 78–89. 10.1016/j.ydbio.2009.06.033. 19576203

[bit70275-bib-0045] Moriwaki, T. , H. Tani , K. Haga , et al. 2023. “Scalable Production of Homogeneous Cardiac Organoids Derived From Human Pluripotent Stem Cells.” Cell Reports Methods 3, no. 12: 100666. 10.1016/j.crmeth.2023.100666. 38113855 PMC10753388

[bit70275-bib-0046] Mozneb, M. , A. Jenkins , S. Sances , et al. 2024. “Multi‐Lineage Heart‐Chip Models Drug Cardiotoxicity and Enhances Maturation of Human Stem Cell‐Derived Cardiovascular Cells.” Lab on a Chip 24, no. 4: 869–881. 10.1039/D3LC00745F. 38252454 PMC12015978

[bit70275-bib-0047] Murphy, S. L. (2024). *Mortality in the United States, 2023*. *521* . 10.15620/cdc/170564 PMC11770397 39819663

[bit70275-bib-0048] Nakajima, Y. 2019. “Retinoic Acid Signaling in Heart Development.” Genesis 57, no. 7–8: e23300. 10.1002/dvg.23300. 31021052

[bit70275-bib-0049] Ng, A. H. M. , P. Khoshakhlagh , J. E. Rojo Arias , et al. 2021. “A Comprehensive Library of Human Transcription Factors for Cell Fate Engineering.” Nature Biotechnology 39, no. 4: 510–519. 10.1038/s41587-020-0742-6. PMC7610615 33257861

[bit70275-bib-0050] Odiete, O. , M. F. Hill , and D. B. Sawyer . 2012. “Neuregulin in Cardiovascular Development and Disease.” Circulation Research 111, no. 10: 1376–1385. 10.1161/CIRCRESAHA.112.267286. 23104879 PMC3752394

[bit70275-bib-0051] Palencia‐Desai, S. , M. S. Rost , J. A. Schumacher , et al. 2015. “Myocardium and BMP Signaling Are Required for Endocardial Differentiation.” Development 142, no. 13: 2304–2315. 10.1242/dev.118687. 26092845 PMC4510589

[bit70275-bib-0052] Pinto, A. R. , A. Ilinykh , M. J. Ivey , et al. 2016. “Revisiting Cardiac Cellular Composition.” Circulation Research 118, no. 3: 400–409. 10.1161/CIRCRESAHA.115.307778. 26635390 PMC4744092

[bit70275-bib-0053] Protze, S. I. , J. H. Lee , and G. M. Keller . 2019. “Human Pluripotent Stem Cell‐Derived Cardiovascular Cells: From Developmental Biology to Therapeutic Applications.” Cell Stem Cell 25, no. 3: 311–327. 10.1016/j.stem.2019.07.010. 31491395

[bit70275-bib-0054] Qu, X. , C. Harmelink , and H. S. Baldwin . 2022. “Endocardial‐Myocardial Interactions During Early Cardiac Differentiation and Trabeculation.” Frontiers in Cardiovascular Medicine 9: 857581. 10.3389/fcvm.2022.857581. 35600483 PMC9116504

[bit70275-bib-0056] Quijada, P. , M. A. Trembley , A. Misra , et al. 2021. “Coordination of Endothelial Cell Positioning and Fate Specification by the Epicardium.” Nature Communications 12, no. 1: 4155. 10.1038/s41467-021-24414-z. PMC8260743 34230480

[bit70275-bib-0057] Radhakrishnan, H. R. G. , P. Chithamparam , S. M. K. Thiagamani , A. Muthukumaran , and N. Ayrilmis . 2025. “Advancing Cardiac Tissue Engineering With Smart Biopolymers: From Fabrication Strategies to 4D Printing and Organoids.” Polymers for Advanced Technologies 36, no. 7: e70280. 10.1002/pat.70280.

[bit70275-bib-0058] Ream, M. W. , L. N. Randolph , Y. Jiang , Y. Chang , X. Bao , and X. L. Lian . 2024. “Direct Programming of Human Pluripotent Stem Cells Into Endothelial Progenitors With SOX17 and FGF2.” Stem Cell Reports 19, no. 4: 579–595. 10.1016/j.stemcr.2024.02.006. 38518781 PMC11096437

[bit70275-bib-0059] Repina, N. A. , H. J. Johnson , X. Bao , et al. 2023. “Optogenetic Control of Wnt Signaling Models Cell‐Intrinsic Embryogenic Patterning Using 2D Human Pluripotent Stem Cell Culture.” Development 150, no. 14: dev201386. 10.1242/dev.201386. 37401411 PMC10399980

[bit70275-bib-0060] Repina, N. A. , T. McClave , H. J. Johnson , X. Bao , R. S. Kane , and D. V. Schaffer . 2020. “Engineered Illumination Devices for Optogenetic Control of Cellular Signaling Dynamics.” Cell Reports 31, no. 10: 107737. 10.1016/j.celrep.2020.107737. 32521262 PMC9357365

[bit70275-bib-0061] Richards, D. J. , Y. Li , C. M. Kerr , et al. 2020. “Human Cardiac Organoids for the Modelling of Myocardial Infarction and Drug Cardiotoxicity.” Nature Biomedical Engineering 4, no. 4: 446–462. 10.1038/s41551-020-0539-4. PMC7422941 32284552

[bit70275-bib-0062] Robinson, A. S. , S. C. Materna , R. M. Barnes , S. De Val , S. M. Xu , and B. L. Black . 2014. “An Arterial‐Specific Enhancer of the Human Endothelin Converting Enzyme 1 (ECE1) Gene Is Synergistically Activated by Sox17, FoxC2, and Etv2.” Developmental Biology 395, no. 2: 379–389. 10.1016/j.ydbio.2014.08.027. 25179465 PMC4252918

[bit70275-bib-0063] Rossi, G. , N. Broguiere , M. Miyamoto , et al. 2021. “Capturing Cardiogenesis in Gastruloids.” Cell Stem Cell 28, no. 2: 230–240.e6. 10.1016/j.stem.2020.10.013. 33176168 PMC7867643

[bit70275-bib-0064] Saba, R. , K. Kitajima , L. Rainbow , et al. 2019. “Endocardium Differentiation Through Sox17 Expression in Endocardium Precursor Cells Regulates Heart Development in Mice.” Scientific Reports 9, no. 1: 11953. 10.1038/s41598-019-48321-y. 31420575 PMC6697751

[bit70275-bib-0065] Sallam, K. , D. Thomas , S. Gaddam , et al. 2022. “Modeling Effects of Immunosuppressive Drugs on Human Hearts Using Induced Pluripotent Stem Cell–Derived Cardiac Organoids and Single‐Cell RNA Sequencing.” Circulation 145, no. 17: 1367–1369. 10.1161/CIRCULATIONAHA.121.054317. 35467958 PMC9472526

[bit70275-bib-0066] Schmidt, C. , A. Deyett , T. Ilmer , et al. 2023. “Multi‐Chamber Cardioids Unravel Human Heart Development and Cardiac Defects.” Cell 186, no. 25: 5587–5605.e27. 10.1016/j.cell.2023.10.030. 38029745

[bit70275-bib-0067] Sheibani, M. , Y. Azizi , M. Shayan , et al. 2022. “Doxorubicin‐Induced Cardiotoxicity: An Overview on Pre‐Clinical Therapeutic Approaches.” Cardiovascular Toxicology 22, no. 4: 292–310. 10.1007/s12012-022-09721-1. 35061218

[bit70275-bib-0068] Silva, A. C. , O. B. Matthys , D. A. Joy , et al. 2021. “Co‐Emergence of Cardiac and Gut Tissues Promotes Cardiomyocyte Maturation Within Human Ipsc‐Derived Organoids.” Cell Stem Cell 28, no. 12: 2137–2152.e6. 10.1016/j.stem.2021.11.007. 34861147

[bit70275-bib-0069] Song, M. , D. B. Choi , J. S. Im , et al. 2024. “Modeling Acute Myocardial Infarction and Cardiac Fibrosis Using Human Induced Pluripotent Stem Cell‐Derived Multi‐Cellular Heart Organoids.” Cell Death & Disease 15, no. 5: 308. 10.1038/s41419-024-06703-9. 38693114 PMC11063052

[bit70275-bib-0072] Tatekoshi, Y. , C. Chen , J. Shapiro , et al. 2024. “Human Induced Pluripotent Stem Cell‐Derived Cardiomyocytes to Study Inflammation‐Induced Diastolic Dysfunction.” eLife 13: RP95867. 10.7554/eLife.95867.1. 39331464 PMC11434618

[bit70275-bib-0073] Voges, H. K. , S. R. Foster , L. Reynolds , et al. 2023. “Vascular Cells Improve Functionality of Human Cardiac Organoids.” Cell Reports 42, no. 5: 112322. 10.1016/j.celrep.2023.112322. 37105170

[bit70275-bib-0074] Volmert, B. , A. Kiselev , A. Juhong , et al. 2023. “A Patterned Human Primitive Heart Organoid Model Generated by Pluripotent Stem Cell Self‐Organization.” Nature Communications 14, no. 1: 8245. 10.1038/s41467-023-43999-1. PMC10716495 38086920

[bit70275-bib-0075] Wang, K. , R. Z. Lin , X. Hong , et al. 2020. “Robust Differentiation of Human Pluripotent Stem Cells Into Endothelial Cells via Temporal Modulation of ETV2 With Modified mRNA.” Science Advances 6, no. 30: eaba7606. 10.1126/sciadv.aba7606. 32832668 PMC7439318

[bit70275-bib-0076] Wang, X. , and P. Yang . 2008. “In Vitro Differentiation of Mouse Embryonic Stem (mES) Cells Using the Hanging Drop Method.” Journal of Visualized Experiments no. 17: e825. 10.3791/825. PMC3253613 19066514

[bit70275-bib-0077] Wauchop, M. , N. Rafatian , Y. Zhao , et al. 2023. “Maturation of Ipsc‐Derived Cardiomyocytes in a Heart‐on‐a‐Chip Device Enables Modeling of Dilated Cardiomyopathy Caused by R222Q‐SCN5A Mutation.” Biomaterials 301: 122255. 10.1016/j.biomaterials.2023.122255. 37651922 PMC10942743

[bit70275-bib-0078] Xia, C. , L. Shao , L. Ma , et al. 2025. “Captopril Alleviates Radiation‐Induced Pulmonary Fibrosis by Suppressing PAI‐1 Expression and Cytoskeleton‐Dependent Epithelial‐To‐Mesenchymal Transition.” European Journal of Pharmacology 1005: 178045. 10.1016/j.ejphar.2025.178045. 40780595

[bit70275-bib-0079] Yang, D. , J. Gomez‐Garcia , S. Funakoshi , et al. 2022. “Modeling Human Multi‐Lineage Heart Field Development With Pluripotent Stem Cells.” Cell Stem Cell 29, no. 9: 1382–1401.e8. 10.1016/j.stem.2022.08.007. 36055193

[bit70275-bib-0080] Yang, J. , W. Lei , Y. Xiao , et al. 2024. “Generation of Human Vascularized and Chambered Cardiac Organoids for Cardiac Disease Modelling and Drug Evaluation.” Cell Proliferation 57, no. 8: e13631. 10.1111/cpr.13631. 38453465 PMC11294415

[bit70275-bib-0081] Ye, C. , C. Yang , H. Zhang , et al. 2024. “Canonical Wnt Signaling Directs the Generation of Functional Human PSC‐Derived Atrioventricular Canal Cardiomyocytes in Bioprinted Cardiac Tissues.” Cell Stem Cell 31, no. 3: 398–409.e5. 10.1016/j.stem.2024.01.008. 38366588

[bit70275-bib-0082] Yi, S. A. , Y. Zhang , C. Rathnam , T. Pongkulapa , and K. B. Lee . 2021. “Bioengineering Approaches for the Advanced Organoid Research.” Advanced Materials 33, no. 45: 2007949. 10.1002/adma.202007949. PMC8682947 34561899

