# Supplementary material for: Cardiovascular Organoids With Adjustable Endothelial Composition via SOX17‐Engineered hPSCs
Source: Biotechnol Bioeng. 2026 Jun 21;123(10):2655–67. doi: 10.1002/bit.70275 (PMC13551498; doi:10.1002/bit.70275)
Supplement: Supplementary file 1 [file BIT-123-2655-s001.docx]

**Cardiovascular Organoids with Adjustable Endothelial Composition via SOX17-Engineered hPSCs**

Po-Yu Liang^1,2,*^, Gyuhyung Jin^1,2,*^, Nathan R Petrucci^3^, Xiaojun Lance Lian^4,5,6#^, Xiaoping Bao^1, 2#^

^1^ Davidson School of Chemical Engineering, Purdue University, West Lafayette, IN, 47907, USA

^2^ Purdue Institute for Cancer Research, West Lafayette, IN, 47907, USA

^3^ Weldon School of Biomedical Engineering, Purdue University, West Lafayette, IN, 47907, USA

^4^ Department of Biomedical Engineering, The Pennsylvania State University, University Park, PA, 16802, USA

^5^ The Huck Institutes of the Life Sciences, The Pennsylvania State University, University Park, PA, 16802, USA

^6^ Department of Biology, The Pennsylvania State University, University Park, PA, 16802, USA

^*^Co-first authors, ^#^Corresponding authors (X.L.L., lian@psu.edu; X. B., bao61@purdue.edu)

**Supplementary Figures**

**Supplemental Figure 1**. Representative immunofluorescence images showing SOX17 expression across varying doxycycline (DOX) concentrations. Yellow = SOX17, blue = Hoechst. Scale bar = 100 μm.

**Supplemental Figure 2**. Representative immunofluorescence and confocal images of cardiac progenitor organoids from embryoid bodies (EBs). Yellow = NKX2.5, blue = Hoechst. Scale bar = 200 μm.

**Supplemental Figure 3**. Representative bright field images of organoids with different H9 to SOX17 cell line ratios (1:2, 1:1, 2:1) between day 6 to day 14. Scale bar = 1 mm.

**Supplemental Figure 4**. **Immunostaining analysis of** **advanced cardiovascular organoids**. (A) Representative cryosection image of advanced cardiac organoids showing discernible internal lumen-like voids (cavities). Scale bar = 500 μm. (B) Representative immunofluorescence and brightfield images of advanced cardiac organoids indicating ring structure of SOX17 signal. Red = SOX17. Scale bar = 100 μm. Blue = Hoechst. Scale bar = 500 μm. (C) Representative cryosection images illustrating the spatial distribution of CMs and ECs in advanced cardiac organoids, with ECs preferentially localized to the interior core and CMs predominantly enriched in the peripheral regions.

**Supplemental Figure 5**. **Representative immunofluorescence images and quantification of MYL7 and MYL2 expression in advanced and innate cardiac organoids**. Yellow = MYL7, green = MYL2, blue = Hoechst. Scale bar = 500 μm. n=5 independent organoids per condition. Data presented as normalized to Hoechst. Values = mean ± SD, Welch’s t-test.

**Supplementary Videos**

**Supplemental Video 1.** Representative immunofluorescence recording of membrane voltage-associated calcium activity, showing dynamic changes in calcium transient intensity during spontaneous contractions of healthy cardiac organoids.

**Supplemental Video 2.** Representative immunofluorescence recording of membrane voltage-associated calcium activity showing reduced calcium transient amplitude in organoids following treatment with 5 μM DXR.

**Supplemental Video 3.** Representative brightfield video of organoid contractile activity before (left) and after (right) isoproterenol (ISO) treatment.
